# Podocalyxin Is a Novel Polysialylated Neural Adhesion Protein with Multiple Roles in Neural Development and Synapse Formation

**DOI:** 10.1371/journal.pone.0012003

**Published:** 2010-08-10

**Authors:** Nathalia Vitureira, Rosa Andrés, Esther Pérez-Martínez, Albert Martínez, Ana Bribián, Juan Blasi, Shierley Chelliah, Guillermo López-Doménech, Fernando De Castro, Ferran Burgaya, Kelly McNagny, Eduardo Soriano

**Affiliations:** 1 Institute for Research in Biomedicine, Parc Cientific de Barcelona, Barcelona, Spain; 2 Centro de Investigación Biomédica en Red sobre Enfermedades Neurodegenerativas (CIBERNED), Barcelona, Spain; 3 Department of Cell Biology, University of Barcelona, Barcelona, Spain; 4 Grupo de Neurobiología del Desarrollo-GNDe, Hospital Nacional de Parapléjicos, Toledo, Spain; 5 Department of Experimental Pathology, Institut d'Investigació Biomèdica de Bellvitge, University of Barcelona, L'Hospitalet de Llobregat, Barcelona, Spain; 6 The Biomedical Research Centre, University of British Columbia, Vancouver, Canada; Hospital Vall d'Hebron, Spain

## Abstract

Neural development and plasticity are regulated by neural adhesion proteins, including the polysialylated form of NCAM (PSA-NCAM). Podocalyxin (PC) is a renal PSA-containing protein that has been reported to function as an anti-adhesin in kidney podocytes. Here we show that PC is widely expressed in neurons during neural development. Neural PC interacts with the ERM protein family, and with NHERF1/2 and RhoA/G. Experiments *in vitro* and phenotypic analyses of *podxl*-deficient mice indicate that PC is involved in neurite growth, branching and axonal fasciculation, and that PC loss-of-function reduces the number of synapses in the CNS and in the neuromuscular system. We also show that whereas some of the brain PC functions require PSA, others depend on PC per se. Our results show that PC, the second highly sialylated neural adhesion protein, plays multiple roles in neural development.

## Introduction

Neural migration and axonal guidance are governed by several families of extracellular cues, which elicit either attractive or repulsive responses on leading edges and axonal growth cones. Prominent members of these families include Netrins and various classes of Semaphorins [1], [2], [3]. In addition, neural development involves cell-to-cell contact and adhesion to the extracellular matrix, which also contribute to the assembly of brain regions and the formation of axonal connections [4], [5]. Adhesion molecules, such as NCAM, L1, or TAG1, have pivotal roles in axonal growth and fasciculation, neural cell migration and synaptogenesis [6], [7], [8], [9], [10]. Moreover, some of these proteins cooperate in signaling events triggered by extracellular factors [6], [11], [12]. In previous studies, we have shown that the highly sialylated renal anti-adhesin Podocalyxin (PC) is expressed in the developing brain [13], [14]. PC is the main glycoprotein expressed on the apical surface of glomerular podocytes. PC is a 140–160 kDa type I transmembrane protein composed of a highly sialylated ectodomain and a short cytoplasmic tail [15], [16]. PC has a strong negative charge and it has been proposed as an anti-adhesin responsible for maintaining the filtration slits open [17], [18]. *podxl*-deficient mice die soon after birth because of defects in kidney development and mutant podocytes do not form foot processes, which leads to glomerular reduced permeability and anuria [19]. PC is also expressed in vascular endothelia, mesothelial cells, hematopoietic stem cells and in several types of tumors [20], [21], [22], [23], [24]. In most circumstances, PC blocks adhesion. In the endothelial venules, however, PC acts as an adhesive ligand for L-selectin-expressing leukocytes [23]. The cytosolic tail may also contribute to the unique organization of podocytes. Two cytosolic adaptor proteins, Na^+^/H^+^-Exchanger Regulatory Factor 2 (NHERF2) and Ezrin, interact with PC in kidney [25]. Given the crucial role of PSA in multiple steps during neural development [26], [27], [28], here we examined the role of PC in brain development. We show that PC is involved in axonal fasciculation and neuritogenesis, and in synaptogenesis.

## Results

### Brain PC is a poly-sialylated protein widely expressed during brain development

**Figure 1:**
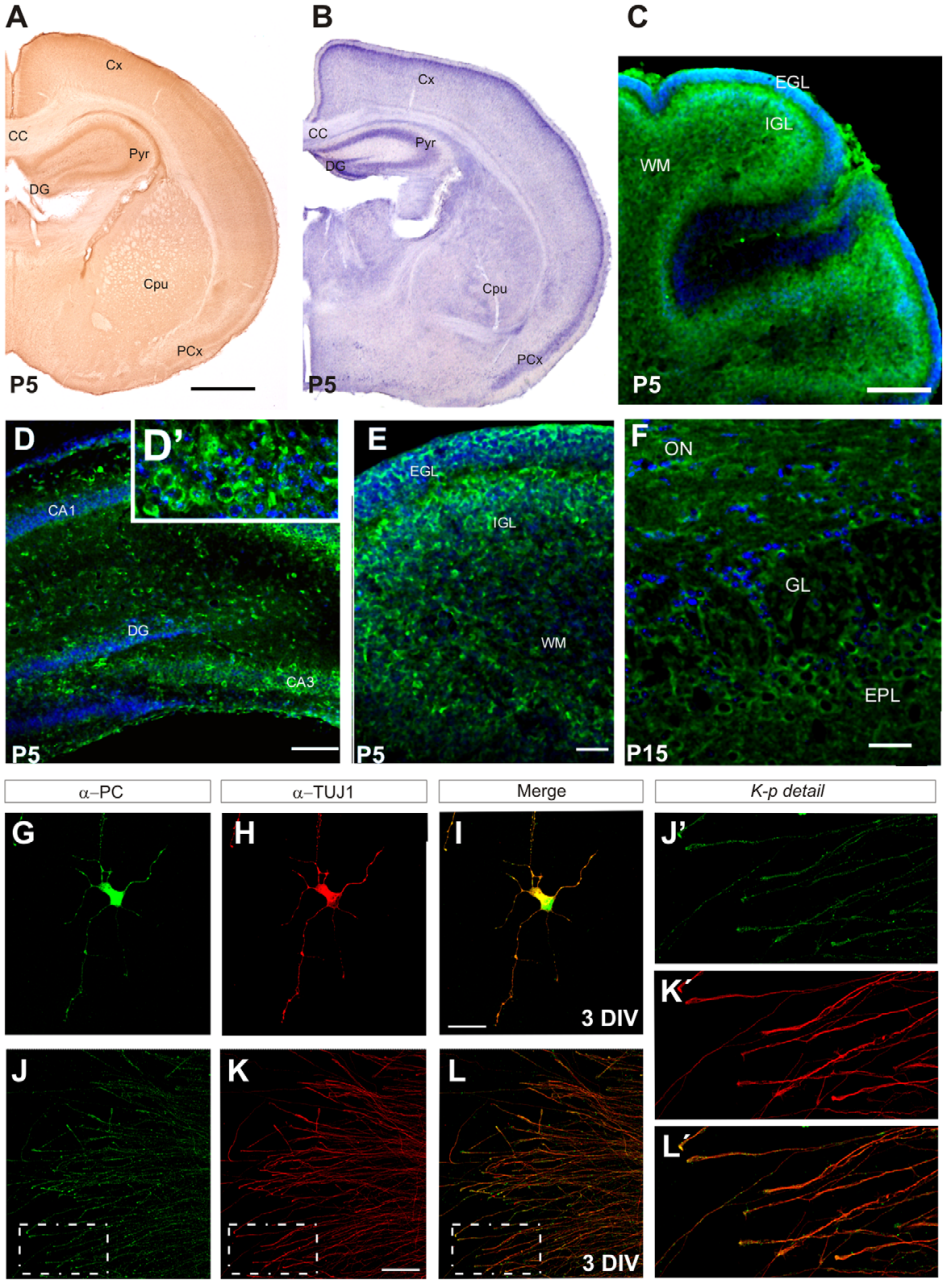
PC is expressed during mouse brain development. (A–C) Distribution of PC mRNA (A) and protein (B–C) at P5. Low-power micrographs of the forebrain (A,B) and cerebellum (C) showing wide expression of PC mRNA and protein in laminar structures and brain nuclei at P5. (D–F) Confocal micrographs illustrating patterns of PC expression in laminated regions, including the hippocampus (D), cerebellum (E) and olfactory bulb (F). Note the pericellular pattern of staining, also evident in the inset in D (D'). (G–L) Distribution of PC protein in developing neurons in culture. Dissociated (G–I) and hippocampal explant cultures (J–L) were incubated with antibodies against PC and the neuronal-specific β-tubulin III (TUJ-1) marker. The merged images are also shown. Neurites (G–I) and axonal processes (J–L) are PC-immunoreactive. Note the punctuate distribution of PC along the hippocampal axons and the localization in axonal growth cones (J'–L'). (J'–L') are magnifications of the boxed areas. CA1, CA3, pyramidal cell regions of the hippocampus; CC, corpus callosum; CPu, caudate-putamen of the striatum; Cx, cerebral cortex; DG, dentate gyrus; EGL, external granular layer; FN, facial nuclei IGL; internal granular layer; PCx, piriform cortex; Pyr, pyramidal cell layer of the hippocampus; P, Purkinje cell layer; WM, white matter; ON, olfactory nerve; GL, glomerular layer; EPL, external plexiform layer. Scale bars: 500 µm (A,B); 125 µm (C); 150 µm (D); 40 µm (E,F); 25 µm (J–L); 20 µm (G–I).

In agreement with a previous study [14], PC mRNA was widely expressed in the developing brain from E12 to adult stages (Fig. 1*a* and Fig. S1). To analyze the fine distribution of PC, we used immunohistochemistry (Fig. 1*b*–*f* and Fig. S1). Two antibodies recognizing the PC extracellular domain (chicken anti-mouse PC and mouse anti-human PC, gift of D. Kershaw; [29]), gave a similar staining pattern to that of the mRNA expression, labeling preferentially cell bodies (Fig. 1*b*–*f* and Fig. S1). In contrast, the rat monoclonal antibody, which also recognizes an extracellular epitope [30], preferentially stained the neuropile and fibers (Fig. S1). Using this antibody, we detected PC in many axonal fascicles throughout the brain during perinatal stages (Fig. S1 *a*). PC mRNA and protein were detected in the proliferative ventricular zones, and especially in postmitotic neurons, including pyramidal cells in the cerebral cortex and hippocampus, periglomerular and granule cells in the olfactory bulb, and Purkinje and granule cells in the cerebellum (Fig. 1*a*–*f* and Fig. S1). Postnatal proliferative zones, such as the EGL in the cerebellum and the SVZ in the forebrain were also intensely labeled (Fig. 1*c,e* and Fig. S1). PC was highly expressed in laminated regions, such as the olfactory bulb, cerebral cortex, hippocampus and cerebellum, and expression was also detected in many nuclei throughout the brain (Fig. 1*a*–*f*, and Fig. S1). Immunostaining of sections from *podxl* ^(−/−)^ brains did not reveal immunolabeling (Fig. S1).

To localize PC with greater precision, hippocampal neuronal cultures were analyzed by immunofluorescence (Fig. 1*g*–*l*). At 2–4 DIV PC immunoreactivity was detected in neuronal cell bodies, dendrites and in axons, including growth cones (Fig. 1*g*–*l*). Hippocampal explants stained with the PC antibodies confirmed that this protein was present in axons and in growth cones (Fig. 1*j*–*l*).At later stages (1–2 weeks *in vitro*) PC protein was in axonal presynaptic bouton-like structures (Fig. S1 *d,h*).

**Figure 2:**
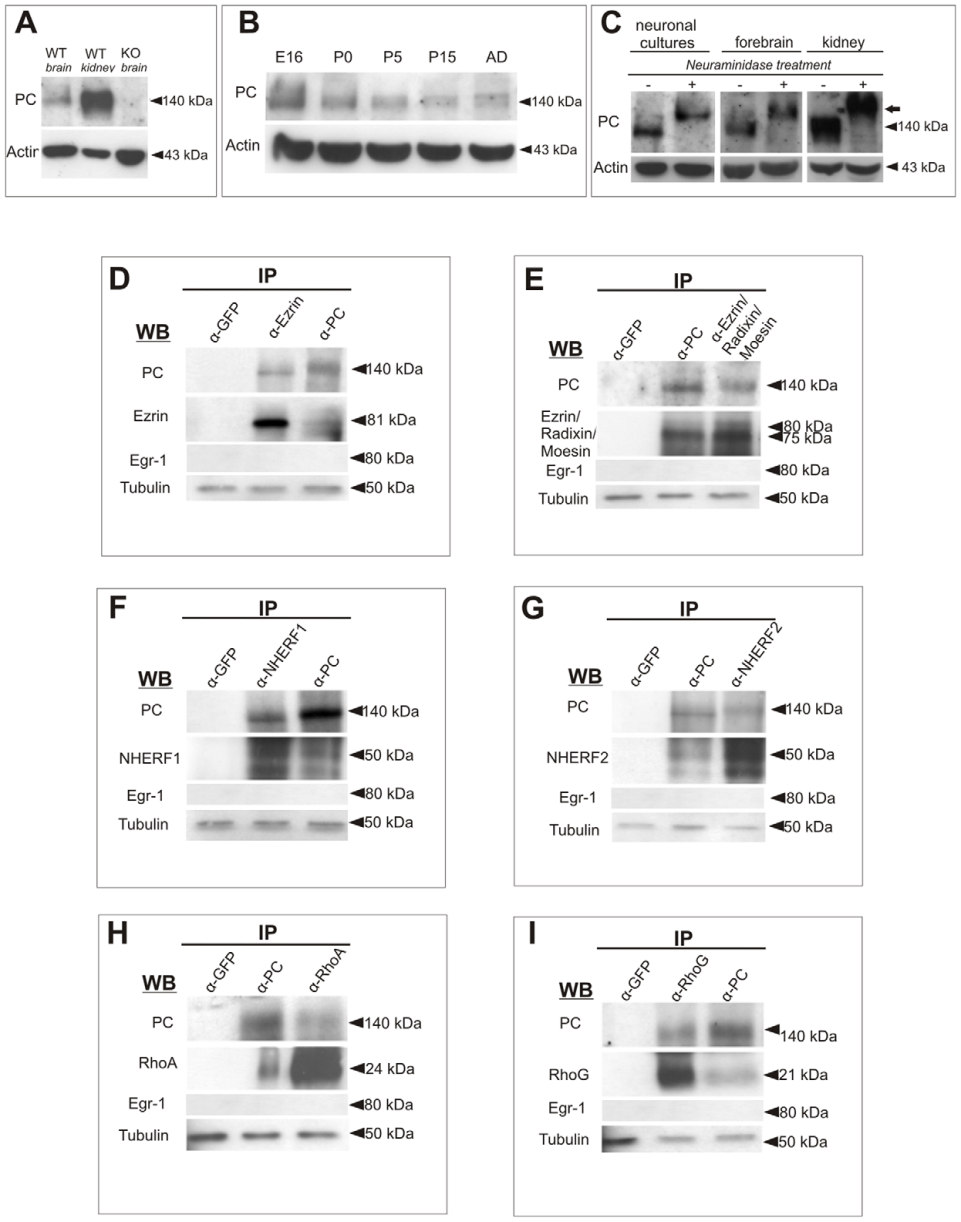
Expression, sialylation and protein interaction of neural PC. (A) Western Blot showing PC immunoreactivity in *wt* brain (E18), kidney (P4) and *podxl* ^−/−^ brain (E18); actin is shown as loading control. (B) Developmental profile of PC-immunoreactivity at several developmental stages; actin immunoreactivity is shown as a loading control. (C) Neuraminidase treatment of hippocampal dissociated cultures (7DIV), E16 brain lysates and adult kidney extracts. Note that the 140-kDa band is sensitive to Neuraminidase digestion in all cases since the mobility of PC is markedly decreased (arrow). (D,E) Ezrin and PC immunoprecipitations of E16 forebrain homogenates. Ezrin immunoprecipitation probed with an α-PC antibody yields co-immunoprecipitation of PC. PC immunoprecipitation yields co-association with Ezrin (D). Co-immunoprecipitation experiments with an antibody recognizing the ERM protein family (Ezrin/Radixin/Moesin) yields identical results (E). (F,G) PC and NHERF1/2 immunoprecipitation assays in forebrain lysates result in co-association of both proteins. (H,I) PC immunoprecipitation results in co-association with the small Rho GTPases RhoA and RhoG; the reverse immunoprecipitation with anti-RhoA/G antibodies also reveals PC protein in the immunoblots. Immunoprecipitation with control, irrelevant antibodies (GFP) did not give positive signals in the immunoblots. Anti-βIII-Tubulin antibodies were used as loading controls.

Expression of PC protein in brain was corroborated by Western Blot with expression peaking at prenatal and early postnatal stages (Fig. 2*a,b*). No band was detected in extracts obtained from *podxl* ^(−/−)^ brains (Fig. 2*a*). In some cases a second, weak PC band was apparent in Western Blots (see Fig. 2*c*). This band was at a similar height as Neuraminidase-treated PC (see below). In kidney, PC is a highly poly-sialylated protein [16], [31]. To assess whether PC is sialylated in brain, extracts of dissociated neuronal cultures and brain lysates were incubated with α2–3, 6, 8, 9-Neuraminidase (Fig. 2*c*). Western Blot analysis revealed that the 140-kDa band is sensitive to Neuraminidase activity since the mobility of PC was decreased (arrow in Fig. 2*c*; Fig. S2), as it is in kidney lysates. This lower mobility may be caused by the lack of negative charges [17]. In contrast, incubation with EndoN did not remove PSA from PC but specifically degraded PSA-NCAM (Fig. S2). This differential degradation supports that PSA in PC is bound by alpha2-6 and alpha2-3 links (O-glycosylation), whereas PSA-NCAM is formed by alpha2-8 links (N-glycosylation) [32].

### Brain PC forms a protein complex with Ezrin, NHERF1/2 and RhoA/G

In kidney and MDCK cells, PC interacts with Ezrin and the sodium-hydrogen exchanger regulatory factor 1 and 2 (NHERF1/2) [25], [33], [34]. To study this interaction in neural tissue, co-immunoprecipitation analyses were performed in E16 brains. When PC antibodies were used to immunoprecipitate lysates, a band of about 80 kDa was detected by Western Blot using anti-Ezrin antibodies (Fig. 2*d*). PC protein was identified in complementary immunoblots from brain lysates immunoprecipitated with anti-Ezrin antibodies (Fig. 2*d*). Experiments using an antibody recognizing the Ezrin/Radixin/Moesin family of proteins gave similar results (Fig. 2*e*). Co-immunoprecipitation experiments also yielded an interaction of brain PC and NHERF1 (Fig. 2*f*). Given that NHERF2 has also been found to be highly expressed in neuronal tissue, we also demonstrated interaction of PC with this additional member of the NHERF family (Fig. 2*g*). Finally, in MDCK cells, PC is linked to small G protein RhoA [34]. Co-immunoprecipation analyses in brain lysates revealed that neural PC also interacts with RhoA and with the related small GTPase RhoG (Fig. 2*h,i*). Controls, including GFP-imunoprecipitation, did not reveal immunolabeling with PC, Ezrin, Ezrin/Radixin/Moesin, RhoA/G or NHERF1/2 antibodies (Fig. 2*d–i*). Further immunoprecipitation controls using WB with an irrelevant antibody (the transcription factor EGR1) did not show signals (Fig. 2*d–i*).

### PC is not required for neuronal migration

**Figure 3:**
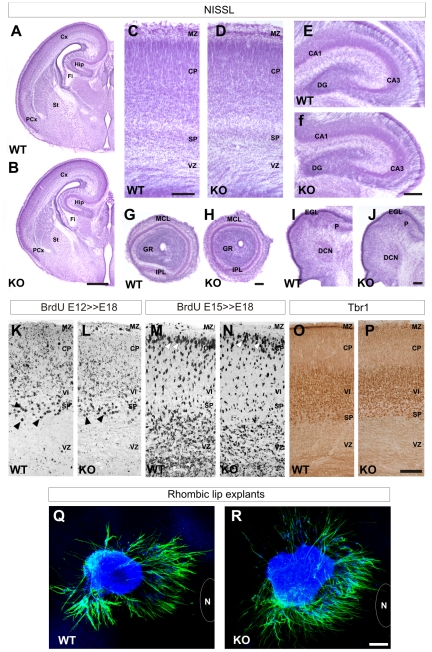
Neuronal migration is not altered in *podxl* ^(−/−)^ embryonic brains. (A,B) Nissl-stained sections showing normal cytoarchitecture in the E18 *podxl* ^(−/−)^ forebrain. Medium power magnifications showing normal cytoarchitecture in the neocortex (C,D), hippocampus (E,F), olfactory bulb (G,H) and cerebellum (I,J) of *podxl* ^(−/−)^ embryos. (K,N) BrdU-immunoreacted sections showing normal distribution of E12- and E15-labeled neurons in the neocortex of *podxl* ^(−/−)^ embryos at E18. E12-labeled BrdU-positive nuclei (arrows) are correctly positioned in the deep cortical layers (K,L). E15-labeled BrdU-positive neurons are positioned in the upper cortical layers (M,N)**.** Cortical layers are indicated to the right. (O,P) Sections immunoreacted with the Tbr1 antibody show normal distribution of upper layer neurons in the neocortex of *podxl* ^(−/−)^ E18 embryos. (Q,R) Explants from the lower rhombic lip co-cultured with aggregates of Netrin-1 expressing cells demonstrate a similar pattern of neurophilic chain migration and chemoattraction in *wt* and *podxl* ^(−/−)^ explants. CA1, CA3, pyramidal cell regions of the hippocampus; CP, cortical plate; Cx, cortex; DCN, deep cerebelar nuclei; DG, dentate gyrus; EGL, external germinative layer; F, fimbria fornix; GR, granular layer of the olfactory bulb; Hip; hippocampus; IPL, internal plexiform layer; MCL, mitral cell layer; MZ, marginal zone; N, netrin-expressing cells; P, Purkinje cell layer; PCx, pyriform cortex SP, subplate; St, striatum; VZ, ventricular zone. Scale bars = 500 µm (A,B); 100 µm (C–F, I–R); 50 µm (G–H).

To analyze the role of PC in brain development, we used *podxl*-deficient mice [19]. Given that these mice die after birth, embryos were analyzed at E18. Nissl-staining of brain sections revealed that the overall organization and cytoarchitecture of *podxl* ^(−/−)^ brains was similar to that of *wt* embryos. Thus, brain regions, nuclei and layers were clearly recognizable throughout the brains of *podxl* ^(−/−)^ embryos, including the layered organization of the cerebral cortex, olfactory bulb and cerebellum, and the nuclear distribution in the thalamus and brain stem (Fig. 3a*–*j). These data suggest that PC is not required for cell proliferation or neuronal migration.

To substantiate these observations, pregnant females were injected with single BrdU-pulses at E12 or E15 and the pattern of radial migration was studied in the neocortex. E12-labeled cohorts were fated to the subplate and layer VI in *wt* and *podxl* ^(−/−)^ embryos (Fig. 3*k–l*); similarly, E15-labeled neurons were properly fated in the upper cortical layers in both genotypes (Fig. 3*m–n*). Correct positioning of migrating neurons in the neocortex was also observed with anti-Tbr1 antibodies (Fig. 3*o,p*), a marker expressed by deep neurons of the cerebral cortex [35]. Taken together, these findings indicate that radial migration is not altered in *podxl*-deficient brains.

To examine the involvement of PC in tangential migration, we analyzed neurophilic chain migration using lower rhombic lip explants (Fig. 3*q,r*). The neurons that migrate from this proliferative region form the circumferential migratory stream, which will give rise to the precerebellar nuclei [36], [37], [38]. In *wt* rhombic lip explants co-cultured with aggregates of Netrin-1-expressing cells, typical chains of migrating neurons were formed that were chemoattracted by Netrin-1-expressing cells (Fig. 3*q,r*). A similar chemoattractive response was observed in *podxl* ^(−/−)^ rhombic lip explants, which showed normal exit of neurons and the formation of migratory chains (Fig. 3*q,r*). We conclude that neither radial nor tangential neuronal migration is impaired by the absence of PC.

### Axonal extension, fasciculation and branching is impaired in PC-deficient neurons

To examine the contribution of PC to axonal growth, E18 brain sections were stained with antibodies against the adhesion proteins L1 and TAG-1 (Fig. S3). Immunostained sections showed that all major axonal tracts were correctly formed in *podxl* ^(−/−)^ brains (Fig. S3, S4). For example, in the forebrain the major commissures (corpus callosum, hippocampal and anterior commissure) and axonal pathways (anterior olfactory tract, reciprocal thalamocortical pathway and cortical white matter) were well developed in these mutant embryos (Fig. S3). Similarly, the topography and radial distribution of fibers was not substantially altered, as seen in the hippocampus and neocortex. Also in the midbrain and hindbrain, all the major axonal tracts in *podxl* ^(−/−)^ brains were distributed normally (not shown). To further analyze axonal targeting in *podxl* ^(−/−)^ embryos, DiI injections were performed in several regions, including the neocortex, olfactory bulb, dorsal thalamus, entorhinal cortex and cerebellum. As illustrated in Fig. S4, cortical injections in the somatosensory cortex labeled corticothalamic fibers which extended through the internal capsule and terminated appropriately in the dorsal thalamus in *wt* and *podxl* ^(−/−)^ brains. Similarly, we found no evidence of aberrant axonal targeting after DiI injections in the remaining brain areas (e.g., the LOT, Fig. S4 *e,f*). These observations indicate that axonal guidance and targeting is not impaired by the lack of PC.

**Figure 4:**
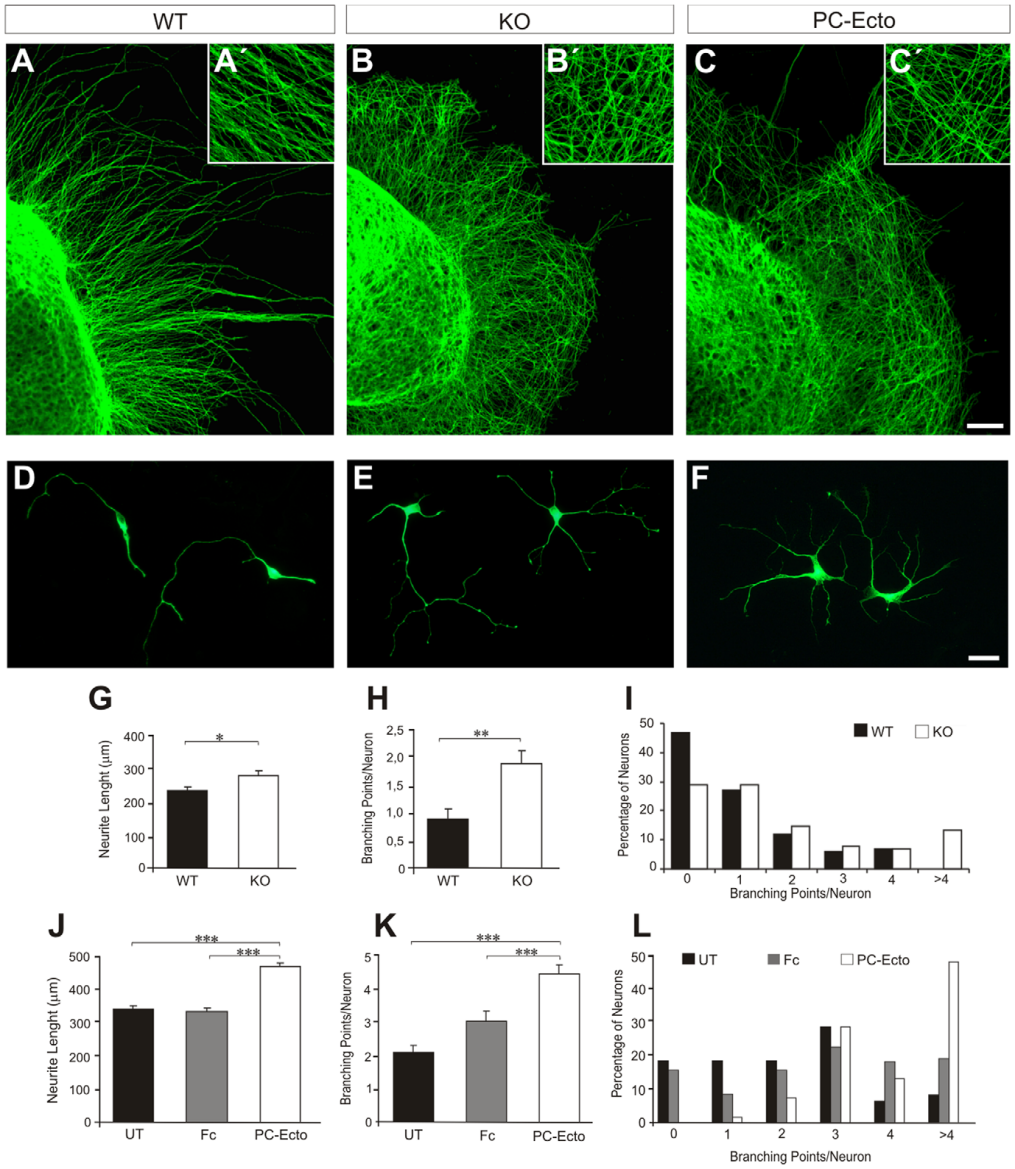
PC regulates neurite outgrowth *in vitro*. (A–C) Examples of *wt* and *podxl* ^(−/−)^ hippocampal explants growing on laminin, and of *wt* explants growing in the presence of PC-Ectodomain. Note the formation of a dense meshwork in PC-deficient conditions. (A'–C') are high-power magnifications of (A–C). Examples of hippocampal *wt* (D) and *podxl* ^(−/−)^ (E) neurons growing on poly-D-lysine, and *wt* neurons growing in the presence of PC-Ectodomain (3DIV) (F). Neurons were labeled with the TUJ-1 antibody. Histograms showing that *podxl* ^(−/−)^ neurons (G, H) and *wt* neurons growing on PC-Ectodomain (J, K) have increased neurite lengths (G, J) and branching points per neuron (H, K), compared to controls. (I, L) Distribution of neurons (per number of branching points) in the different conditions. UT, untreated control; Fc, control substrate with rabbit Fc; PC-Ecto, PC-ectodomain substrate. Mann-Whitney test, statistical significance, * P<0.05, ** P<0.01, *** P<0.001. Data are expressed as mean ± s.e.m. Scale bar = 25 µm (A–C); 15 µm (D–F).

A closer examination of axonal tracts, however, revealed that the shape and size of axonal fascicles differed. Thus, in the mutant hippocampus axonal bundles in the white matter were smaller, less compact and occupied a wider zone in the adjacent *stratum oriens* than those in *wt* littermates (Fig. S3). Similarly, other axonal tracts, such as the habenulo-peduncular tract in the dorsal thalamus (Fig. S3) or the fornix, displayed reduced fasciculation. These data indicate that PC is involved in neuron-to-neuron adhesion and in the fasciculation of developing axonal tracts. Next, we examined the role of PC in axonal growth *in vitro*. Hippocampal *wt* explants gave rise to numerous axons that fasciculated and grew along straight courses (Fig. 4*a*). The pattern of axonal outgrowth in *podxl* ^(−/−)^ explants differed dramatically: axons grew aberrantly following sinusoidal trajectories with little axonal fasciculation and with profuse branching (Fig. 4*b*). Overall, the pattern of axonal growth appeared as a dense meshwork of crisscross fibers. An identical phenotype was observed when *wt* explants were cultured on coverslips coated with soluble PC-ectodomain, which blocks the PC function (Fig. 4*c*).

To substantiate these findings, dissociated hippocampal neurons from *wt* and *podxl* ^(−/−)^mice were cultured and neurite length and the number of branching points per neuron were calculated (Fig. 4*d, e, g–i*). *podxl* ^(−/−)^ neurons exhibited increased neuritogenesis, extended neuritis and a two-fold increase in the number of branching points per neuron compared to *wt* neurons (Fig. 4g–i). Similar results were observed when *wt* hippocampal neurons were cultured on a PC ectodomain substrate, used as a PC blocking reagent, in comparison with control cultures incubated with Fc protein (Fig. 4*f and j–l*). These findings indicate that PC is involved in axonal elongation, fasciculation and axonal branching.

### The lack of PC impairs synaptogenesis

**Figure 5:**
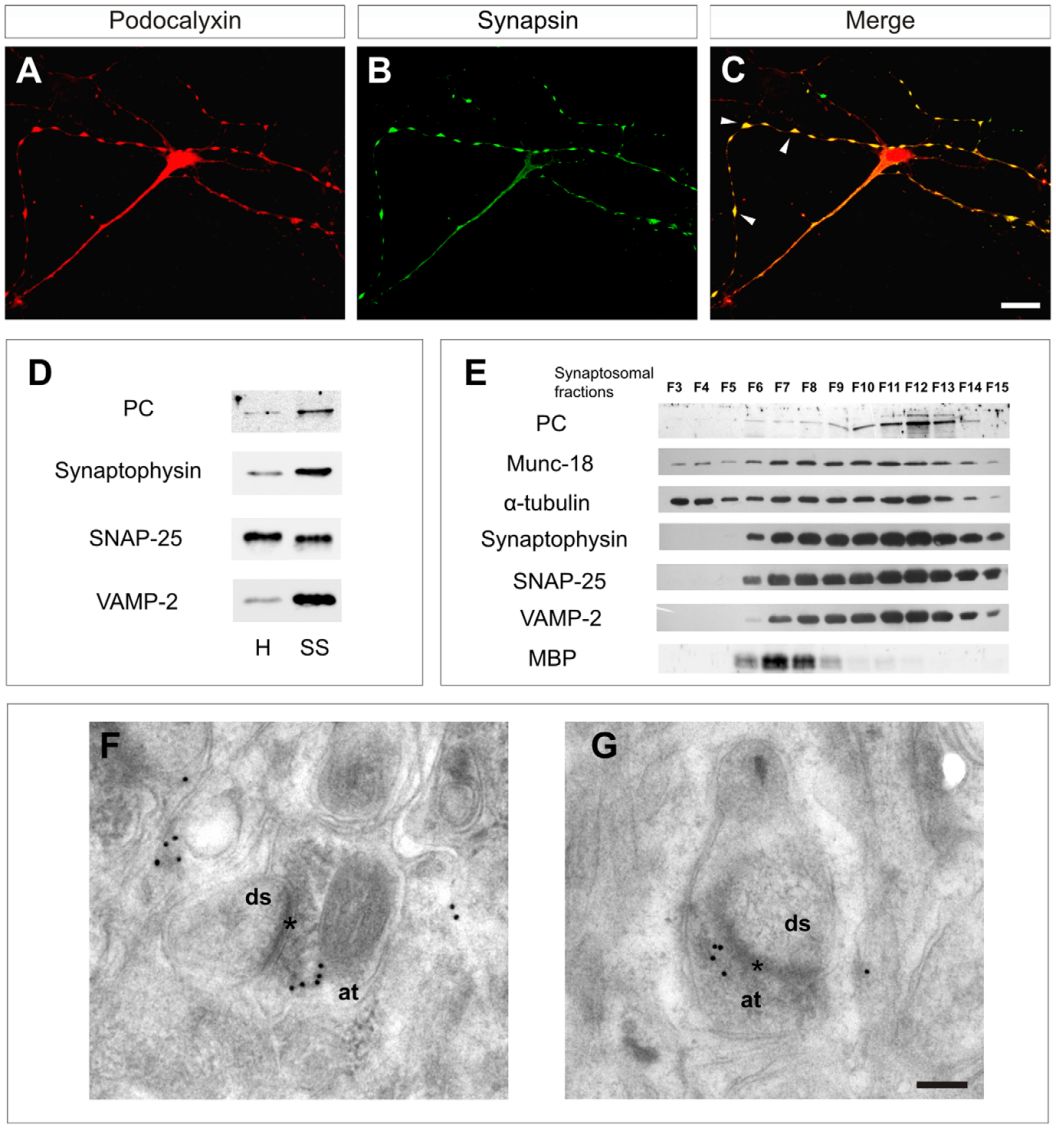
Expression of PC in presynaptic terminals. (A,C) Hippocampal cultures (7DIV) were incubated with antibodies against PC (A) and against Synapsin (B). The merged images are shown in (C). (D) Western Blot showing enriched expression of PC in adult forebrain synaptosomes (SS), in comparison to total brain homogenates (H). Western blots for synaptic vesicles (Synaptophysin and VAMP-2) and axonal membranes (SNAP-25) are shown. (E) Purified synaptosomes were subfractionated in a sucrose gradient. Fractions were collected and analyzed by immunoblot. Note that PC is detected in the fractions enriched in synaptic vesicle markers (F10–F13), whereas it is absent from cytosolic fractions (F3–F5) and expressed at low levels in membrane fractions (F6–F9). Immunoblots for several synaptic markers and for Tubulin are shown. Immunoblot for Myelin Basic Protein is also shown as Myelin is present in the fractions enriched in membranes. (F-,G) Electron micrographs showing localization of PC in presynaptic terminals in the adult hippocampus using post-embedding immunogold labeling. at, axonal terminal; ds, dendrite spine. Scale bars = 15 µm (A–C); 0.25 µm (F,G).

**Figure 6:**
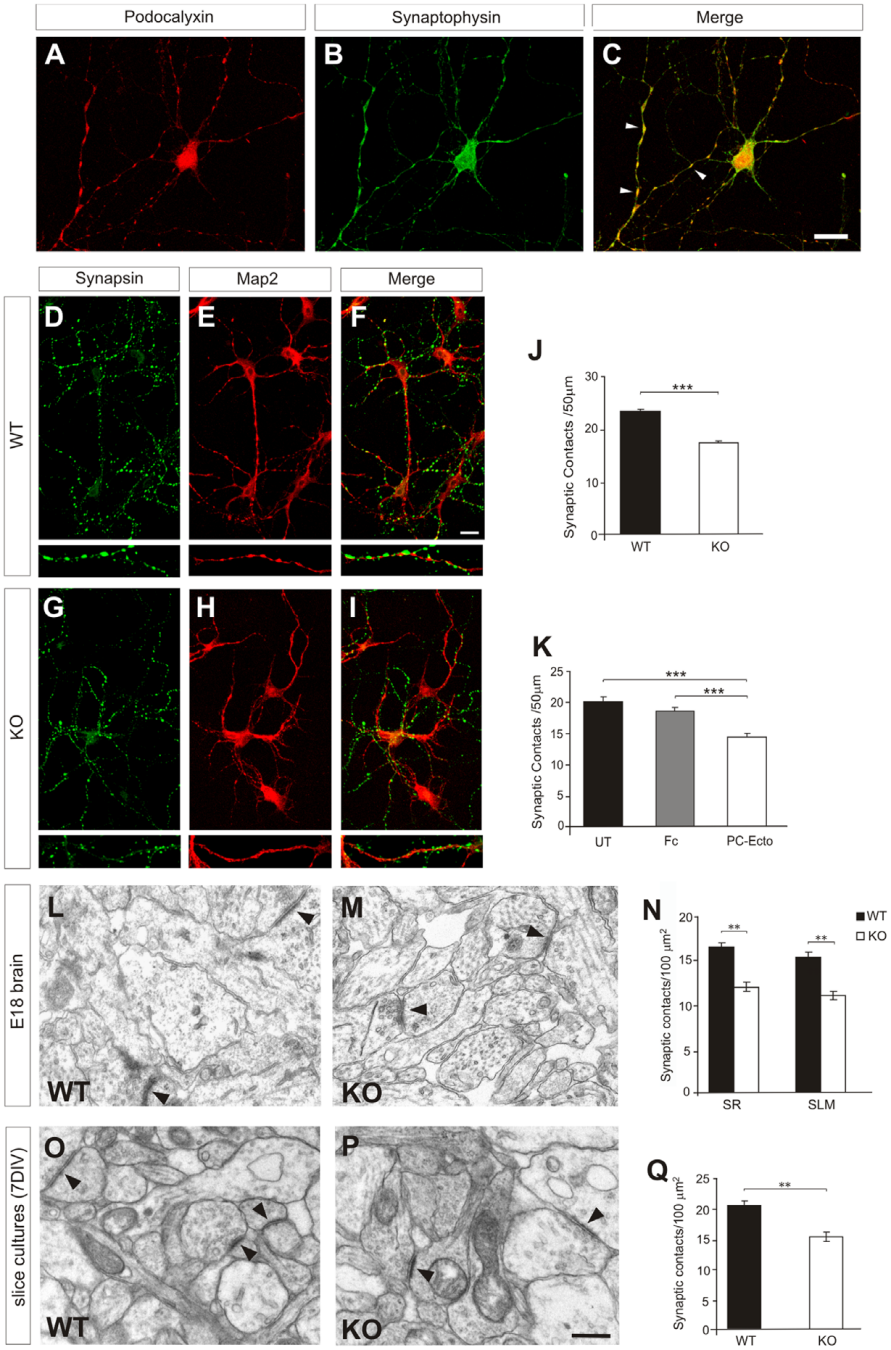
PC is required for correct synaptogenesis in the hippocampus. (A–C) PC expression in presynaptic terminals. Hippocampal cultures (7DIV) were incubated with antibodies against PC (A) and against synaptophysin (B). The merged image is shown in (C). (D–I) Hippocampal neuronal cultures from *wt* and *podxl* ^(−/−)^ embryos immunolabelled for Synapsin I and II and the specific dendritic marker MAP2, showing synaptic appositions on MAP2-positive dendrites. (J,K) Histograms showing that cultures of *podxl* ^(−/−)^ neurons and of *wt* neurons treated during 7DIV with the PC-Ectodomain have a decreased number of synaptic appositions compared to controls. (L-M) Examples of electron micrographs illustrating hippocampal synaptic contacts (arrows) in *wt* and *podxl* ^(−/−)^ E18 hippocampi. (N) Density of synaptic contacts in the *stratum radiatum* and the *stratum lacunosum moleculare*, showing decreased number of synapses in *podxl* ^(−/−)^ E18 embryos. (O,P) Electron micrographs showing synaptic contacts (arrowheads) in *wt* and *podxl* ^(−/−)^ E18 hippocampal slice cultures, cultured for 7 additional DIV. (Q) Density of synaptic contacts in hippocampal organotypic cultures, showing decreased number of synapses in *podxl* ^(−/−)^ slices. UT, untreated control; Fc, rabbit Fc containing medium; PC-Ecto, PC ectodomain-containing medium; SR, *stratum radiatum*; SLM, *stratum lacunosum moleculare.* Mann-Whitney test (J,K) or Student's *t* test (N,Q) were used; statistical significance, ** P<0.01, *** P<0.001. Data are expressed as mean ± s.e.m. Scale bars = 10 µm (A–I); 0.5 µm (L,M,O,P).

We next examined whether PC participates in synapse formation. First, PC localization was analyzed in 7–10 DIV hippocampal cultures. PC was enriched in axonal-like varicosities, where PC co-localized with synaptic proteins such as Synaptophysin and Synapsin I and II (Fig. 5*a–c* and 6*a–c*). To confirm that PC was expressed in presynaptic terminals, we prepared adult synaptosomes and analyzed the presence of PC protein by Western Blot. PC was enriched in adult synaptosomal (6-fold) compared to total brain homogenates (H, total homogenates; and SS, synaptosomal fraction; in Fig. 5*d*). Further fractionation of synaptosomal preparations through a sucrose gradient showed that PC was enriched in the vesicular fractions (F10*–*F13, displaying Synaptophysin, VAMP2 and SNAP25 immunoreactivities) compared to the cytosolic (F3*–*F5, tubulin) and membrane (F6*–*F9, MBP immunoreactivity) fractions (Fig. 5*e*). Finally, immunogold techniques were used to localize PC at the fine structural level. Hippocampal dendrites and postsynaptic dendritic spines were devoid of immunolabeling. In presynaptic axon terminals gold particles were preferentially associated with synaptic vesicles (Fig. 5*f,g*). These data indicate that PC is enriched in developing and mature presynaptic axon terminals in the adult brain.

To determine the possible involvement of PC in synaptogenesis, we first studied the formation of synaptic-like appositions *in vitro* (Fig. 6*d–i*). Hippocampal cultures (7DIV) were immunostained with MAP2 and with the presynaptic marker Synapsin I and II, and presynaptic appositions over MAP2-positive dendrites were counted. *podxl* ^(−/−)^ neurons showed about a 25% decrease in the density of presynaptic-like structures apposed to MAP2-immunolabeled dendrites (Fig. 6*j*). A similar decrease was observed when *wt* neurons were incubated with the blocking PC ectodomain protein, but not with control Fc protein (Fig. 6*k*). We next examined synaptic development in E18 brains *in vivo* by electron microscopy (Fig. 6*l,m*). Presynaptic terminals containing a few synaptic vesicles were identified in the embryonic hippocampus in *wt* and *podxl* ^(−/−)^ embryos. The density of synaptic contacts was decreased by 23% in the *stratum lacunosum moleculare* and by 25% in the *stratum radiatum* of the hippocampus of the latter compared to *wt* littermates (Fig. 6*n*). *podxl*-deficient mice do not survive after birth, therefore to ascertain whether decreased synaptogenesis is transient or persistent, we prepared organotypic hippocampal slices from E18 embryos that were cultured for 7 DIV. Electron microscopy examination did not show changes in the morphology of synaptic contacts between both genotypes. Again, the density of synaptic contacts was decreased (by 28%) in the hippocampus of the former (Fig. 6*o–q*).

**Figure 7:**
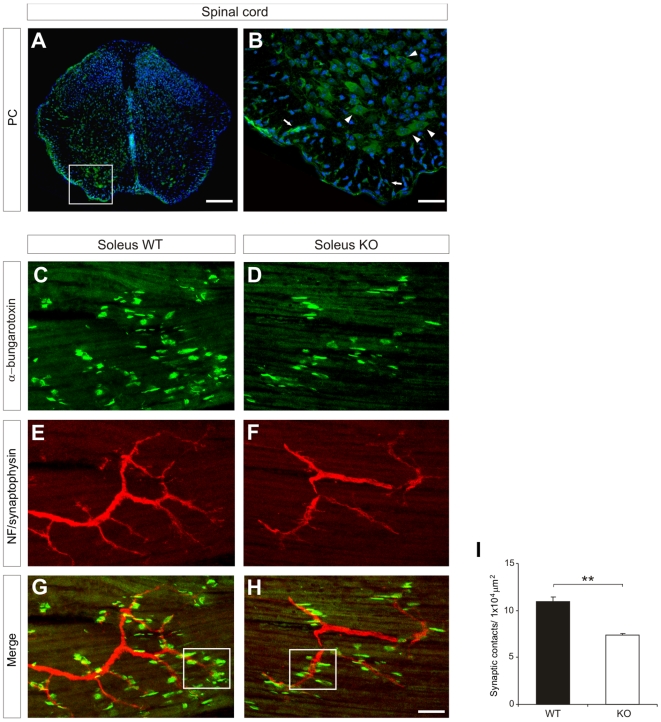
*podxl* ^(−/−)^ embryos show a reduction in the density of AChR clusters in the neuromuscular system. (A, B) Traversal sections of the spinal cord (E18) showing PC-immunoreactivity in motorneurons. Sections counterstained with bisbenzimide. (C–G) Longitudinal slices of soleus muscles from *podxl* ^(−/−)^ and *wt* littermates at E18, stained with Alexa488-α-bungarotoxin to visualize postsynaptic AChRs (C, D) and antibodies against neurofilament 200 and synaptophysin (E, F) to visualize motor axons and nerve terminals. The merged images are shown in (G, H). Open squares in G and H illustrate sample areas used for the quantitative determination of synaptic junctions. (I) Bar graph showing that *podxl* ^(−/−)^ soleus muscles have a decreased density of AChR clusters compared to controls. The Student's *t* test was used; statistical significance, ** P<0.01. Scale bars = 150 µm (A); 50 µm (C–H); 40 µm (B).

Because PC mRNA and protein were also expressed in motorneurons (Fig. 7a,b), we analyzed the role of PC in the formation of neuromuscular synapses. Sections from the soleus muscle were stained with α-bungarotoxin (to stain cholinergic receptors and thus synaptic junctions) and anti-Neurofilament 200/Synaptophysin antibodies (staining axons) to label post- and pre-synaptic terminals respectively. The density of neuromuscular synaptic specializations in 1×10^4^ um^2^ surface samples (open squares in Fig. 7*d,g*) was calculated. Neuromuscular synapses were decreased by 32% in *podxl* ^(−/−)^ E18 embryos compared to *wt* littermates (Fig. 7*c–h*). These findings implicate PC protein in synaptogenesis in the central and peripheral nervous system.

### PSA-dependent and -independent functions of PC in neural development

**Figure 8:**
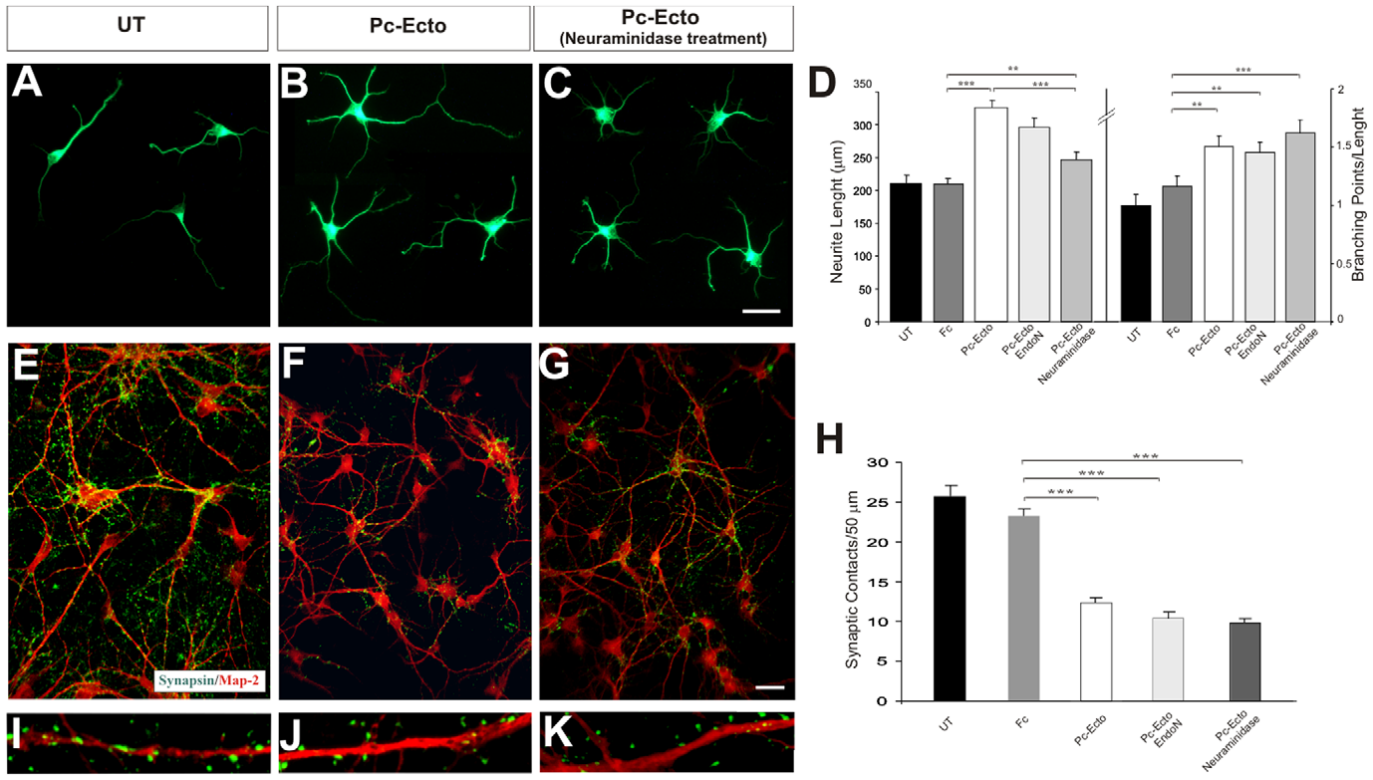
PSA-dependent and PSA-independent functions of PC. (A–C) Examples of hippocampal neurons cultured in control medium (A), or in the presence of PC-Ectodomain (B) or PC-Ectodomain incubated with neuraminidase (C). Neurons were labeled with the TUJ-1 antibody. (D) Histograms showing that whereas the effects on neurite length are blocked by Neuraminidase treatment (left histogram), the effects on branching remain unchanged after Neuraminidase treatment (right histogram). (E–G) Hippocampal neuronal cultures immunolabelled for Synapsin I/II and the specific dendritic marker MAP2, showing synaptic appositions on MAP2-positive dendrites, in control conditions (E), and after treatment with PC-Ectodomain(F) or with PC-Ectodomain incubated with Neuraminidase (G). (I–K) illustrate high-power confocal micrographs. (H) Histogram showing that cultures of hippocampal neurons show decreased number of synaptic appositions after incubation with either PC-ectodomain or PC-Ectodomain treated with Neuraminidase. UT, untreated control; Fc, control substrate with rabbit Fc; PCc-Ecto, PC-ectodomain substrate; PCc-Ecto/EndoN, PC-Ectodomain substrate incubated with EndoN; PC-Ecto/Neuraminidase, PC-Ectodomain substrate incubated with Neuraminidase. The Student's *t* test was used; statistical significance, * P<0.05, ** P<0.01 and *** P<0.001. Scale bars = 15 µm (A–C), 10 µm (W–F), 30 µm (L–N).

To determine whether the functions of brain PC are dependent on PSA or on PC per se, we carried out *in vitro* experiments by incubating cells with PSA-PC ectodomain or with this ectodomain treated with Neuraminidase (non-sialylated PC). We also used EndoN (cleaving specifically PSA-NCAM) as a control (Fig. 8). We addressed whether axonal phenotypes depended on PSA-bound PC. In hippocampal neurons, the increase in neurite length induced by PSA-PC was blocked when PSA was removed from PC (Fig. 8*a–d*). In contrast, the increased neuronal branching seen in PSA-PC treated cultures remained unchanged upon removal of PSA (Fig. 8*a–d*). Treatment with EndoN did not result in modifications. These findings suggest that while PSA-PC is required for the neurite growth phenotype, non-sialylated PC is responsible for the branching phenotype.

Using a similar approach, we also found that both sialylated and non-sialylated forms of PC induced an identical decrease in hippocampal synaptogenesis (Fig. 8*e–k*), thereby indicating that PC per se (and not PSA) is responsible for this reduction. Taken together, our results strengthen the notion that the neurite extension function of PC is mediated by PSA carried by the protein, while non-sialylated PC is responsible for neurite branching and synapse formation phenotypes.

## Discussion

Cell adhesion molecules play an essential role during several developmental stages in neural tissue [7], [39]. PSA-NCAM has a pivotal role in these functions [7], [40]. Here we show that PC is widely expressed throughout CNS development and in the adult brain, and that it is highly sialylated in neuronal tissue. The presence of a second, weak PC band, particularly enriched in synaptosomal fractions, suggests that both sialylated and non-sialylated forms of PC are expressed in brain. To our knowledge, PC is the second highly-sialylated adhesion glycoprotein expressed in neurons, in addition to NCAM. Here we show that the lack of PC function results in increased axonal elongation, branching and synaptogenesis. Furthermore, our study demonstrates the PC effects on branching and synaptogenesis do not require PSA, but appear to be dependent on PC protein alone. The highly negatively charged PSA-PC is believed to act as an anti-adhesive protein by a charge repulsion mechanism in kidney podocytes [25], [41]. Such an anti-adhesive role is consistent with the increased neurite length phenotype, which is blocked after PSA removal. Other PC phenotypes, however, particularly in processes that are independent of PSA (axonal branching and synaptogenesis), are unlikely to respond to an anti-adhesive function, but correlate better with an adhesive role of PC. However, the putative PC ligands that mediate brain adhesion remain to be identified.

The highly polysialylated form of the NCAM is required for the proper tangential migration of interneuron precursors along the Rostral Migratory Stream (RMS). Mice deficient for all the splice variants [42] or for only the 180-kDa isoform [10] show a reduction in olfactory bulb size and an accumulation of migrating neurons along the RMS. At early postnatal stages, this effect is phenocopied by genetic or enzymatic removal of PSA, thereby indicating the relevance of PSA modification in this process [27], [43]. In the present study we found no defects either in the olfactory bulb or in the RMS of *podxl* ^(−/−)^ embryos. This observation indicates that, at least in prenatal stages, PC is not essential for chain migration in the RMS. In contrast, NCAM is not required for radial migration in laminated brain regions, such as the cerebral cortex, the cerebellum or the olfactory bulb *in vivo* [10], [42]. Interestingly, ST8SiaII and ST8SiaIV double-mutant mice show reduced radial migration in the neocortex [26], thereby implicating an additional PSA-containing protein in radial migration. As our phenotypic studies in *podxl* ^(−/−)^ embryos did not show radial migration alterations, NCAM and PC may have redundant functions in neuronal radial migration. One of the most dramatic alterations produced by the lack of PC function in neurons is enhanced growth and branching of neurites and axons. This finding suggests that PC is a negative regulator of neurite branching, at least in embryonic stages. Although neurite branching is crucial to development, its mechanisms are not fully understood. A number of adhesion and extracellular molecules, including Netrins, Semaphorins and BDNF regulate neurite branching [44], [45], [46]. In contrast, RhoA is a negative regulator of dendrite outgrowth in various organisms and cell types [47], [48]. In kidney podocytes PC forms a complex with Ezrin and the NHERF2, which promotes RhoA activation and stabilizes the actin cytoskeleton [25], [34]. In the present study, we found that brain PC interacts with the Ezrin/NHERF1-2/RhoA-G complex, thereby suggesting that activation of this protein complex is responsible for the reduction in neurite growth and branching observed in PC loss-of-function models.

The cell adhesion molecule NCAM has been shown to act as a co-receptor for the neurotrophic factor GDNF [12]. A recent study has demonstrated that in the hematopoietic system Podocalyxin co-associates with the SDF-1 receptor CXCR4 thus modulating the biochemical signaling response to this cytokine [49]. It is therefore possible that part of the functions of Podocalyxin in neural development observed in the present study are related to the above molecular mechanism, i.e., modulation of neurotrophic factor and cytokine signaling by this novel adhesion protein.

Deficiency of NCAM, and other adhesion molecules such as L1, often results in axonal defasciculation [9], [50], [51]. *ncam*-deficient mice, for instance, show defasciculation of mossy fibers in the hippocampus [50] and pathfinding errors and reduced fasciculation of the corticospinal tract [52]. Interestingly, defects in mossy fiber lamination and major axonal tracts, including malformations in the anterior commissure, corticospinal tract and the corpus callosum, are more dramatic in ST8SiaII and ST8SiaIV double-mutant mice lacking PSA [26], [53]. These observations suggest that, in addition to NCAM, PSA activity is essential for the formation and fasciculation of axonal tracts. Our findings show that PC controls axonal fasciculation *in vitro*. Since the phenotype of *podxl* ^(−/−)^embryos is relatively subtle *in vivo* (though dramatic *in vitro*), our results suggest compensatory mechanisms in *podxl* ^(−/−)^ embryos, possibly caused by PSA-NCAM expression, thereby suggesting functional redundancy of PC and PSA-NCAM in axonal guidance and fasciculation. PSA-NCAM is essential for neural plasticity [54], [55], [56]. *In vitro* experiments show that PSA-NCAM is involved in axonal target selection and stabilization of the synapse [40], [57]. However, *ncam* ^(−/−)^ mice do not show differences in the number of CNS and PNS synapses [57], [58]. Fasciclin II, the NCAM homologue in *Drosophila* [59], and the Aplysia cell adhesion molecule (apCAM) [60] have an essential role in synapse formation, which suggests functional genetic redundancy in mammals. A recent study, however, provides evidence that the removal of PSA-NCAM from the cerebral cortex at postnatal stages leads to a selective precocious maturation of GABAergic synapses [61]. This observation implies that PSA-NCAM may play synaptogenic functions in specific neural populations and developmental ages. Here we report that the lack of PC results in fewer synaptic contacts both *in vivo* and *in vitro* in the CNS and in the neuromuscular system, thereby suggesting that PC is required for the correct formation or stabilization of synapses. In neural development, signals that trigger increased axonal branching and elongation, such as BDNF, often lead to an increased number of synapses [62]. This evidence suggests that the increase in synapses is secondary to longer axonal lengths. However, in our study, the increment of axonal branches in PC loss-of-function models (expected to be associated with increased synaptogenesis) produced an opposite effect, i.e. reduced number of synapses, thereby supporting a direct role of PC in synapse formation or maintenance. Given that the lack of PSA does not affect overall synapse numbers [40], the role of PC in this process may be independent of PSA. Our experiments showing that either PSA-PC or non-sialylated PC triggers identical decreases in synaptogenesis support this view (Fig. 7*h*).

In summary, here we demonstrate that, in addition to PSA-NCAM, the PSA-containing PC protein plays pivotal roles in several processes during early brain development, including neurite outgrowth and branching, fasciculation and synaptogenesis. These developmental processes may be mediated by an NHERF/Ezrin/RhoA pathway, linking PC and the actin cytoskeleton. Our results indicate that PC has a dual anti-adhesive/adhesive role in successive steps of neural development, which are likely to be mediated by highly sialylated and non-sialylated forms of the protein. Thus, our data provide evidence for a role of the Sialomucin/CD34 protein family in the development of the nervous tissue.

## Materials and Methods

All procedures were performed in accordance with the guidelines approved by the Spanish Ministry of Science and Technology and following the European Community Council Directive 86/609 EEC.

### Animals

PC-deficient embryos (*podxl* ^(−/−)^) were obtained and genotyped as described previously [19]. OF1 embryos and postnatal mice (Iffra Credo, Lyon, France) were used in this study. The mating day was considered as embryonic day 0 (E0) and the day of birth as postnatal day 0 (P0). For OF1, the following developmental stages were studied: E12, E14, E16, E18, P0, P5, P10, P15, P21, and adult (three to five animals for each stage). Animals were anesthetized with 4% halothane. Since *podxl* ^−/−^ mice die within 24 h of birth, embryos were used. *Wt* embryos of the same littermates were used as controls in all the experiments.

### Antibodies

Primary antibodies were used against PC (chicken, 1∶250, gift by Dr. David Kershaw, University of Michigan Medical Centre, USA; rat monoclonal, 1∶1000, R&D System, Minneapolis, USA; mouse anti-human PC 1∶100, [29]), BrdU (rat monoclonal, 1∶100, Harlan Sera-Lab, Loughborough, UK), Tbr-1 (rabbit polyclonal, 1∶200, gift from Dr. Carlos Vicario, CSIC, Spain), neuronal specific β-III Tubulin (TUJ-1, mouse monoclonal, 1∶4000, Babco, Richmond, VA), A2B5 (mouse monoclonal, 1∶50, Hybridoma Bank, Iowa, USA), GFP (rabbit polyclonal, 1∶500, Molecular Probes, Eugene, USA), SNAP-25 (SMI81, mouse monoclonal, 1∶1000, Becton-Dickinson, USA), Syntaxin 1 (mouse monoclonal, 1∶1000, Hybridoma Bank, Iowa, USA), Synaptophysin (mouse monoclonal, 1∶1000, Dako Diagnostics S.A), VAMP-2 (mouse monoclonal 1∶1000, Synaptics System, USA), Synapsin (rabbit polyclonal, 1∶1000, Synaptics System, USA), Neurofilament 200 kD (mouse monoclonal, 1∶300, Sigma-Aldrich, St Louis, MO), TAG-1 (rabbit polyclonal, 1∶1000, gift from Dr. Fritz G. Rathjen, Max-Delbrück-Centrum, Berlin, Germany), PSA-NCAM (mouse IgM, 1.1000; AbCys, Paris, France), L1 (rabbit polyclonal, 1∶1000, gift from Dr. Fritz G. Rathjen, Max-Delbrück-Centrum, Berlin, Germany), Actin (mouse monoclonal, 1∶1000, Chemicon, Temecula, CA), and MAP2 (mouse monoclonal, 1∶200, Babco, Richmond, VA).

### PC ectodomain-Fc preparation

DNA encoding the Fc of rabbit IgG was cloned into the 5′-Pst and 3′-XhoI sites of the pSecTag2A vector (Invitrogen, Carlsbad, CA). The cDNA corresponding to the extracellular domain from mouse PC were generated by PCR and cloned 5′of the Fc to produce PC ectodomain-Fc fusion protein. The PC ectodomain fusion construct was expressed in EBNA-293 cells for 2–3 days following transfection with Lipofectamine Plus (Invitrogen, Carlsbad, CA). The supernatant was collected, concentrated, filtered and maintained at 4°C for dissociated and explant culture treatment.

### Immunohistochemistry and *in situ* hybridization

Embryos and postnatal mice were perfused transcardially with 4% paraformaldehyde. Thereafter, the brains were cryoprotected and frozen on dry ice. Coronal sections (thickness: E14-E18: 50 µm, P0-adult: 30 µm) were obtained and processed for immunohistochemistry. Sections were washed in PBS and PBS-Triton X-100, blocked for 2 h and incubated with primary anti-PC antibodies diluted in blocking solution, overnight at 4°C. The sections were then incubated with secondary biotinylated antibodies (Vector Laboratories, Inc., Burlingame, CA) in blocking solution for 2 h and with a streptavidin-horseradish peroxidase complex (Amersham Pharmacia Biotech). Sections were developed with 0.03% diaminobenzidine and 0.002% hydrogen peroxide, mounted on slides, dehydrated, and coverslipped. Sections were also stained by immunofluorescence. Incubation with non-immunized IgGs and omission of primary antibodies prevented immunostaining. *In situ* hybridization was performed on free-floating sections as described previously [14].

The soleus muscles of E18 *podxl^(^* ^−/−)^ and control littermates were dissected. The muscles were cryoprotected in 30% sucrose-PBS and frozen on dry ice. Longitudinal sections (thickness: 50 µm) were obtained with a Leica CM 1325 cryostat and cryoprotected in a solution containing 30% glycerol, 30% ethylene glycol, 40% 0.1 M (PBS), and stored at −30°C until use. For immunohistochemistry, muscle sections were processed as described above. FITC-conjugated bungarotoxin (10^−8^ M, Molecular Probes, Eugene, USA) was added with secondary antibodies overnight at 4°C. Sections were recorded in a Leica TCS 4D laser scanning confocal microscope (Leica Lasertechnik). For quantitative analysis of AChR clusters, the numbers of AChR clusters in a matching 1×10^4^ µm^2^ area from control (n = 3) and mutant (n = 3) soleus were counted.

### Post-embedding immunocytochemistry

OF1 adult mice (n = 2) were perfused with 4% PFA-0.1% glutaraldehyde in 0.12 M phosphate buffer. Brains were removed and small samples of hippocampus were dissected, cryoprotected gradually in sucrose and cryofixed by immersion in isopentane. Freeze-substitution was performed at −90°C over 3 days in an “Automatic Freeze Substitution System” (AFS, Leica), using methanol containing 0.5% uranyl acetate as substitution medium. Brains were infiltrated in Lowicryl HM20 at −50°C and then polymerized with UV lamps. Ultrathin sections were collected and processed for post-embedding PC immunostaining using a chicken anti-PC antibody (1∶25), and biotinylated rabbit anti-chicken (1∶100; R&D System) and 15 nm colloidal gold-coated secondary antibodies (BBI; 1∶25). In control experiments, the primary PC antibody was omitted. No immunogold labeling occurred in these conditions.

### Electron microscopy

E18 mutants (n = 3) and control (n = 3) littermates were perfused with 1% glutaraldehyde–1% paraformaldehyde in 0.12 M phosphate buffer. Brains were fixed in the same solution overnight. Tissue slices were post-fixed with 2% osmium tetroxide, stained with 2% uranyl acetate and embedded in Araldite. Ultrathin sections were collected on formvar-coated slot grids and stained with lead citrate. Electron micrographs covering 64 µm^2^ (final magnification 20,000×) were randomly taken from the *stratum radiatum* and the *stratum lacunosum-moleculare* of the hippocampus, and the number of synaptic contacts was counted (n = 50–77 micrographs per layer and group).

In addition, hippocampal slice cultures were prepared from E18 *podxl* ^(−/−)^ mice and control littermates (n = 2 embryos per group) as described [63]. Mice were anesthetized by hypothermia, their brains were removed, and the hippocampal formations were dissected out. Horizontal sections (350 µm thick) were obtained using McIlwain tissue chopper. Selected slices were cultured using the interphase membrane method [64]. After 7 DIV, hippocampal cultures were fixed in 1% glutaraldehyde–1% paraformaldehyde in 0.12 M phosphate buffer and processed for electron microscopy (see above). Ultrathin sections of the *stratum radiatum* from two hippocampi per animal type were obtained and electron micrographs covering 64 µ^2^ (n = 42 per group) were randomly taken and the density of synaptic (both symmetric and asymmetric) contacts was analyzed.

### DiI tracing

For tracing developing connections, the neocortex, dorsal thalamus, olfactory bulb, entorhinal cortex and cerebellum of E18 embryos were injected with a small crystal of the lipophilic tracer DiI (1,1′ dioctadecyl-3,3,3′,3′ tetramethylindocarbocynanine; Molecular Probes, Eugene, USA). Three *podxl* ^(−/−)^ and three *wt* counterparts were used for these studies. After some weeks in fixative, vibratome coronal sections were stained with bisbenzimide and viewed under epifluorescence.

### 5′-Bromodeoxyuridine studies

For the 5′-bromodeoxyuridine (BrdU) experiments, time-pregnant *podxl* ^(+/−)^ females were injected with a single BrdU pulse (50 mg/kg) at E12 or E15 (n = 2 females per age). After perfusion with paraformaldehyde at E18, littermate brains were fixed in Carnoy's solution, embedded in paraffin, and sectioned coronally at 10 µm. After DNA denaturation, sections were immunolabeled for BrdU as described [65], using a DAB-nickel-enhanced reaction and coverslipped.

### Protein brain extracts and Western blot

The brains were collected at E16, E18, P0, P5, P15 and adult. In brief, the forebrains were homogenized in 20 mM HEPES (pH 7), 150 mM NaCl, 5 mM EGTA, 1 mM MgCl_2_, 10% glycerol, 1 mM aprotinin, 1 mM leupeptin, 0.2 mM PMSF, 0.1 M NaF, 10 mM sodium pyrophosphate, and 0.2 mM sodium orthovanadate. After centrifugation at 9000 *g* for 1 min, supernatants were analyzed by Western Blot. Aliquots of 50 µg of protein were treated with α2-3, 6, 8, 9-Neuraminidase (Sigma-Aldrich, St Louis, MO) or EndoN (Abcys) at 37°C for 1–3 h following the manufacturer's instructions.

Samples were loaded and run in polyacrylamide gels at 100 V. After running, transfer to nitrocellulose membranes was performed in 120 mM glycine, 125 mM Tris, 0.1% SDS, and 20% methanol, 10% mercaptoethanol. Transfer was performed at 35 V ON. Filters were then blocked in 5% powder milk in TBS and incubated with primary antibodies (anti-PC or -PSA-NCAM). Secondary antibodies were used diluted 1∶2500 in TBS containing 5% powder milk. Labeling was visualized with ECL plus (Amersham Pharmacia Biotech).

### Immunoprecipitations

E16 forebrains were lysed using a buffer containing 50 mM HEPES, 150 mM NaCl, 1.5 mM MgCl_2_, 1 mM EGTA, 1% Triton-100, 10% glycerol, 1 mM NaF, 0.5 M sodium pirophosphate and 200 mM ortoguanadate. 300 µg of total protein per sample was used for the immunoprecipitation assays. Homogenates were incubated with α-PC (1∶250, MBL and 1∶250, R&D), α-Ezrin (1∶50, Thermo Scientific), α-Ezrin/Radixin/Moesin (1∶50, Cell Signalling Technology), α-NHERF1, α-NHERF2 (both 1∶500, gifts from Dr. Chris Yun), α-RhoA (1∶50, Santa Cruz Biotechnology), α-RhoG (1∶50, Santa Cruz Biotechnology) or α-GFP (1.500, Invitrogene). All the assays were performed using protein G-Sepharose beads (Sigma) for 2 h at 4°C. After five washes with the washing buffer (10 mM Tris pH 8, 500 mM NaCl, 1 mM EDTA, 1 mM EGTA, 1% Triton X-100, 0.5% NP-40), SDS-sample buffer was added to the beads and they were then boiled at 95°C for 5 min. Proteins were analyzed by SDS-PAGE and Western blot. After SDS-PAGE, proteins were transferred onto Nitrocellulose membranes, which were blocked with 5% non-fat dry milk in Tris-HCl-buffered saline (TBS) containing 0.1% Tween 20, and incubated overnight at 4°C with antibodies against PC (1∶1000), Ezrin (1∶5000), Ezrin/Radixin/Moesin (1∶1000), NHERF1 (1∶7500), NHERF2 (1∶4500), RhoA (1∶50, Santa Cruz Biotechnology), RhoG (1∶50, Santa Cruz Biotechnology) or EGR1 (1∶1000, Santa Cruz Biotechnology). Tubulin was used as loading control. After incubation with the appropriate HPR-conjugated secondary antibodies, blots were developed following the ECL method (Amersham Pharmacia Biotech).

### Neuronal primary cultures and immunocytochemistry

E16 mouse brains were dissected in PBS containing 0.6% glucose and the hippocampus were dissected out. After trypsin (Invitrogen, Carlsbad, CA) and DNase treatment (Roche Diagnostics), tissue pieces were dissociated by gentle sweeping. Cells were then counted and seeded onto poly-D-lysine-coated coverslips in neurobasal medium containing 1% horse serum and B27 supplement (Invitrogen, Carlsbad, CA). Cells were cultured for periods of 2–10 days. Coverslips were fixed in 2% paraformaldehyde. After fixation, cells were permeabilized with Triton X-100 in PBS and blocked with 10% fetal bovine serum-albumin (Roche Diagnostics) in PBS. To determine whether hippocampal neurons express PC, we labeled the cultures with the chicken anti-PC (1∶200) and anti-TUJ-1 (1∶4000). To study the localization of PC in neurons, we labeled the cultures with chicken anti-PC or rat anti-PC and anti-Synaptophysin (1∶1000), anti-VAMP-2 (1∶1000), anti-SNAP-25 (1∶1000), anti-Syntaxin (1∶1000), anti-Synapsin (1∶1000) or anti-MAP2 in PBS with 5% foetal bovine serum for 2 h and with secondary antibodies labeled with either TRITC or FITC (Alexafluor 568 or 488, Invitrogen, Carlsbad, CA).

For neurite length and branching analysis, 2–3 day-old *wt*, *podxl* ^(−/−)^ and *wt* primary cultures incubated with conditioned medium containing PC ectodomain (30–50 µl conditioned medium in 450 µl of total medium) or with control medium (rabbit Fc) were fixed and labeled with anti-TUJ-1. Quantification of axonal length from hippocampal cells was performed using IMAT software (developed by the Technical Services of the University of Barcelona). For quantification of neurite length and branch number, fixed cells were viewed at 20× magnification. There were about 80 neurons per group. Neurite length was measured from the cell body to the distal end of the process. Total neurite length is the sum of all primary neurites and branches produced by a single neuron. Branching points are the points at which a neurite extends from another neurite. Branching number is the sum of each branching point from a single neuron. Primary neurites are those that extend directly from the soma. To count synaptic contacts, 7-day-old *wt*, *podxl* ^(−/−)^ and *wt* primary cultures incubated with conditioned medium containing PC ectodomain (30–50 µl conditioned medium in 450 µl of total medium) or control medium (rabbit Fc) were fixed and labeled with the MAP-2 and Synapsin I and II antibodies. The density of synaptic appositions was counted. A total of 56–75 neurons per group, from at least 3 separate experiments, were counted.

### Synaptosome subfractionation

Adult mouse forebrains were homogenized in 30 ml of SolA buffer (320 mM sucrose, 5 mM Na-HEPES/HCl, pH 7.4) with ten strokes at 600 r.p.m. in a glass-Teflon homogenizer. The homogenate was centrifuged (5000 r.p.m., SS34, for 5 min at 4°C). The resulting postnuclear supernatant was centrifuged twice at 11000 r.p.m. (SS34, for 12 min at 4°C) and the crude synaptosomal fraction was resuspended in 4–8 ml of SolA buffer. This sample was layered on top of a discontinuous Ficoll gradient of 12%/9%/5%. After centrifugation for 35 min at 22500 r.p.m in an SW28 rotor (Beckman), the synaptosomes were collected at the 5%–9% and 9%–12% interphases and resuspended in 15 ml of sodium buffer (10 mM glucose, 5 mM KCl, 140 mM NaCl, 5 mM NaHCO_3_, 1 mM MgCl_2_, 1.2 MM Na_2_HPO_4_ and 20 mM Hepes/NaOH, pH 7.4). After centrifugation for 12 min at 11000 r.p.m, the pellet was resuspended in 300 µl of sodium buffer and 2.7 ml H_2_O. This sample was layered on top of a discontinuous sucrose gradient (0.4 M, 0.6 M, 0.8 M, 1.0 M, 1.2 M, 1.4 M, 1.6 M, 1.8 M). Gradients were centrifuged for 3 h at 33000 r.p.m in an SW41 rotor (Beckman) and were collected in 0.5 ml fractions. The enrichment of the fractions was assessed with several markers by immunoblotting (Synaptophysin, SNAP25, VAMP2, Munc18, Tubulin, MBP).

### Explant Cultures

*Wt* and *podxl* ^−/−^ explants from the E16 CA3 hippocampal region, the E13 lower rhombic lip (lRL) and E16 optic nerve were used in this study. Hippocampal explants were cultured for 3 days on a laminin substrate and were stained with the TUJ1 antibody, which was visualized with immunofluorescence. For function-blocking analysis, *wt* hippocampal explants were cultured on a substrate with laminin and the PC ectodomain (1∶2). After 2–3 days of culture, the explants were labeled as described above.

lRL explants of *wt* and *podxl* ^(−/−)^ embryos were co-cultured with aggregates of Netrin 1-expressing 293T cells or aggregates of control 293T cells for 2 days in a Matrigel matrix (Becton Dickinson). Explants were stained with TUJ-1 mAb and bisbenzimide (Sigma-Aldrich, St Louis, MO) and visualized by immunofluorescence.

### Neuraminidase and EndoN treatments in cultures

The PC ectodomain-Fc fusion construct (or its control rabbit Fc) was expressed and concentrated as above. Aliquots of PC-ectodomain were incubated with Neuraminidase or EndoN for 1–3 h (see above). Western Blots with anti-PC or PSA-NCAM antibodies were routinely run to assess the PC sialylation patterns. Dissociated hippocampal cultures were prepared as above and cultured for either 3 DIV or 7 DIV in the following conditions: untreated with PC, control substrate with rabbit Fc, PC-ectodomain substrate, PC-Ectodomain substrate incubated with EndoN or PC-Ectodomain substrate incubated with Neuraminidase. Cultures were fixed and stained with the TUJ-1 antibody or with the MAP2/Synaptohysin antibodies as above.

### Statistical analysis

Data were analyzed with the program Statgraphics Plus 5.1 using the ANOVA and the Student's *t* test or the Mann Whitney (W) test. Minimal statistical significance was fixed at p<0.05. In Figures, * indicates P<0.05, ** indicates P<0.01 and *** indicates P<0.001. Data are expressed as mean ± s.e.m.

## Supporting Information

Figure S1 PC is widely expressed in laminated brain regions during development. (A–D) Immunohistochemical labeling in the hippocampus at P0 (A) and P5 (B,D). Pattern of PC mRNA expression in the hippocampus at P5 (C). The pattern of PC-immunoreactivity with the rat monoclonal PC antibody shows intense staining in fibers in the alveus (arrowheads) and fimbria at P0 (A), and punctate staining in the neuropile at P5 (D). The labeling seen with the chicken antibody (B) matches that of mRNA expression. (E–N) Sections showing PC protein (E,F,H,I,J,L,N) and mRNA (G,K,M) expression at several developmental stages in the neocortex (E–H), third ventricle (I), olfactory bulb (J,K) and cerebellum (L–N) at different ages. (O,P) Hippocampal sections from wt and podx(−/−) littermates showing absence of PC immunolabeling in sections from PC-deficient embryos. CA1, CA3, pyramidal cell regions of the hippocampus; DG, dentate gyrus; MZ; marginal zone; CP, cortical plate; IZ, intermediate zone; VZ, ventricular zone; ML, molecular layer; I–VI, cortical cell layers; IIIV, third ventricle; ON, olfactory nerve; GL, glomerular layer; EPL, external plexiform layer; EGL, external granular layer; P, Purkinje cell layer; DCN, deep cerebellar nuclei EGL; EPL, external plexiform layer; GL, glomerular cell layer; IGL, internal immature granular layer of the cerebellum. Scale bars = 100 µm (E–I); 75 µm (L); 50 µm (I, O,P); 30 µm (J,K,N).
(7.45 MB TIF)

Figure S2 Sialylation of PC protein in brain. (A) Western Blot showing PC and NCAM immunoreactivities in E16 brain lysates in control conditions (UT), and after treatment with EndoN and Neuraminidase. Neuraminidase removes PSA from PC, but not from NCAM; in contrast, EndoN removes PSA from NCAM, but not from PC. (B) Western Blot of brain extracts from different ages treated with neuraminidase shows that PC is sialylated in both developing and adult stages.
(6.66 MB TIF)

Figure S3 PC-deficient embryos display normal axonal trajectories but abnormal fasciculation. (A, B) Low-power views of L1-immunolabeled forebrain sections showing a normal distribution of fibers in podx(−/−) embryos, compared to wt brains at E18. (C–F), shape and size of TAG-1 immunoreactive axonal fascicles are altered in PC-deficient hippocampus. Note that in the podx(−/−) hippocampus (E, F) axonal bundles of the white matter were smaller, less compacted and occupied a wider zone in the adjacent stratum oriens, compared to wt littermates (C, D). (D, F) high magnification of C and E, respectively. (G, H) TAG1-immunoreacted sections showing increased defasciculation in the habenulo-peduncular tract of podx(−/−) embryos. Cx, cerebral cortex; CPu, caudate-putamen of the striatum; Hip, hippocampus; hpt, habenulo-peduncular tract; T, thalamus. Scale bars = 400 µm (A, B), 200 µm (C, E); 50 µm (D, F), 75 µm (G, H).
(10.07 MB TIF)

Figure S4 PC embryos display normal axonal projection patterns. Pattern of DiI labeling in wt (A,B,C) and podx(−/−) embryos (C,D,F) following DiI injections in the cerebral cortex (A,D) and olfactory bulb (E,F). After DiI injections in the cortex, robust corticothalamic projections invade the thalamus in wt (A,B) and podx(−/−) (D,E) embryos, traversing the striatum (Cpu). Arrowheads point to the anterior commissure. (C,F) Labeling of the Lateral Olfactory Tract after DiI injections in the Olfactory Bulb, suggesting normal formation of this tract in podx(−/−) embryos. All images correspond to coronal sections. Cpu, caudate-putamen; LOT, lateral olfactory tract; T, thalamus. Scale bars = 500 µm (A–D); 100 µm (E,F).
(3.94 MB TIF)
