# Supplementary figures and images for: Podocalyxin Is a Novel Polysialylated Neural Adhesion Protein with Multiple Roles in Neural Development and Synapse Formation
Source: PLoS One. 2010 Aug 10;5(8):e12003. doi: 10.1371/journal.pone.0012003 (PMC2919383; doi:10.1371/journal.pone.0012003)

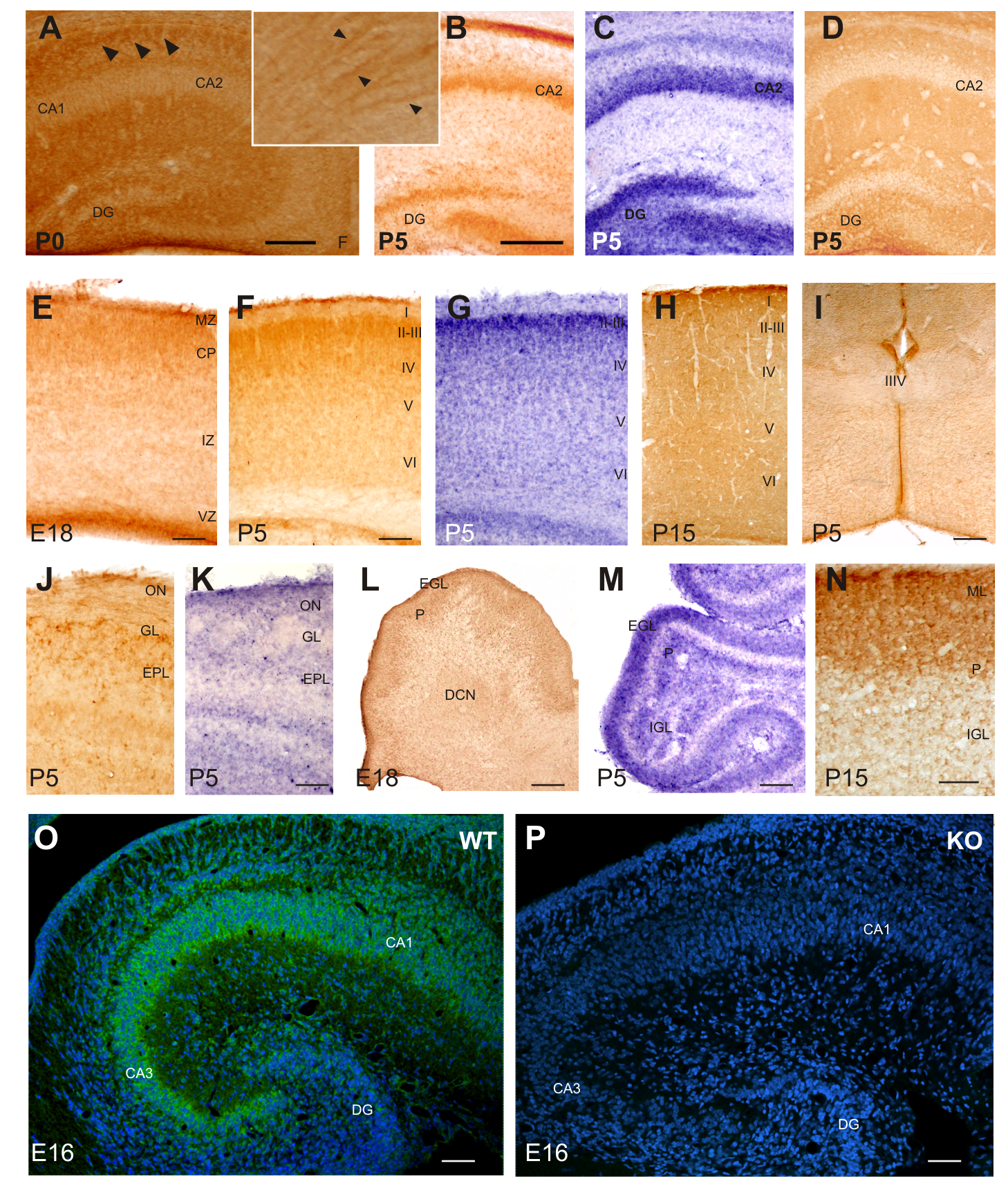

Supplement: Figure S1 — PC is widely expressed in laminated brain regions during development. (A–D) Immunohistochemical labeling in the hippocampus at P0 (A) and P5 (B,D). Pattern of PC mRNA expression in the hippocampus at P5 (C). The pattern of PC-immunoreactivity with the rat monoclonal PC antibody shows intense staining in fibers in the alveus (arrowheads) and fimbria at P0 (A), and punctate staining in the neuropile at P5 (D). The labeling seen with the chicken antibody (B) matches that of mRNA expression. (E–N) Sections showing PC protein (E,F,H,I,J,L,N) and mRNA (G,K,M) expression at several developmental stages in the neocortex (E–H), third ventricle (I), olfactory bulb (J,K) and cerebellum (L–N) at different ages. (O,P) Hippocampal sections from wt and podx(−/−) littermates showing absence of PC immunolabeling in sections from PC-deficient embryos. CA1, CA3, pyramidal cell regions of the hippocampus; DG, dentate gyrus; MZ; marginal zone; CP, cortical plate; IZ, intermediate zone; VZ, ventricular zone; ML, molecular layer; I–VI, cortical cell layers; IIIV, third ventricle; ON, olfactory nerve; GL, glomerular layer; EPL, external plexiform layer; EGL, external granular layer; P, Purkinje cell layer; DCN, deep cerebellar nuclei EGL; EPL, external plexiform layer; GL, glomerular cell layer; IGL, internal immature granular layer of the cerebellum. Scale bars = 100 µm (E–I); 75 µm (L); 50 µm (I, O,P); 30 µm (J,K,N). (7.45 MB TIF) [file pone.0012003.s001.tif]

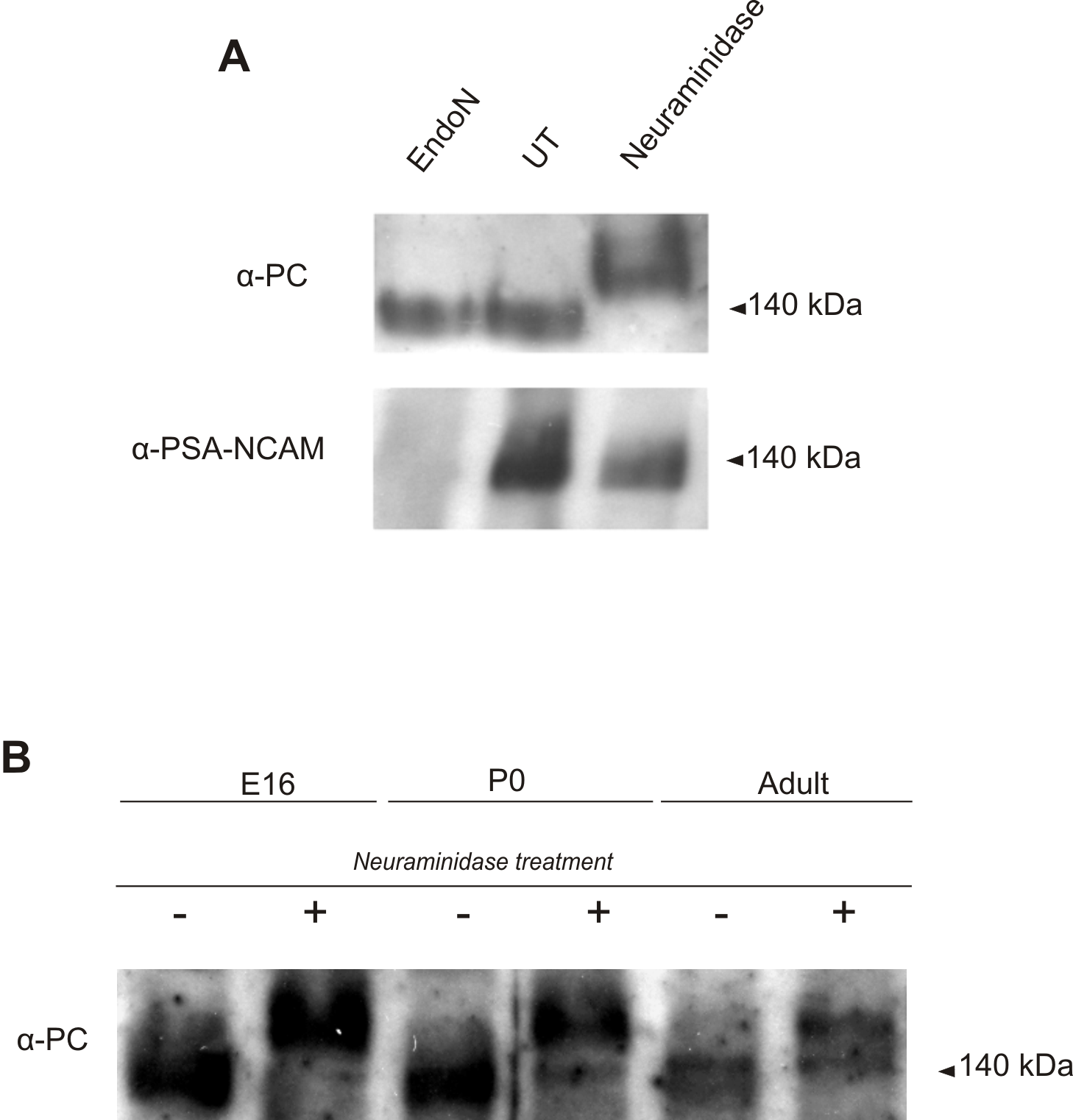

Supplement: Figure S2 — Sialylation of PC protein in brain. (A) Western Blot showing PC and NCAM immunoreactivities in E16 brain lysates in control conditions (UT), and after treatment with EndoN and Neuraminidase. Neuraminidase removes PSA from PC, but not from NCAM; in contrast, EndoN removes PSA from NCAM, but not from PC. (B) Western Blot of brain extracts from different ages treated with neuraminidase shows that PC is sialylated in both developing and adult stages. (6.66 MB TIF) [file pone.0012003.s002.tif]

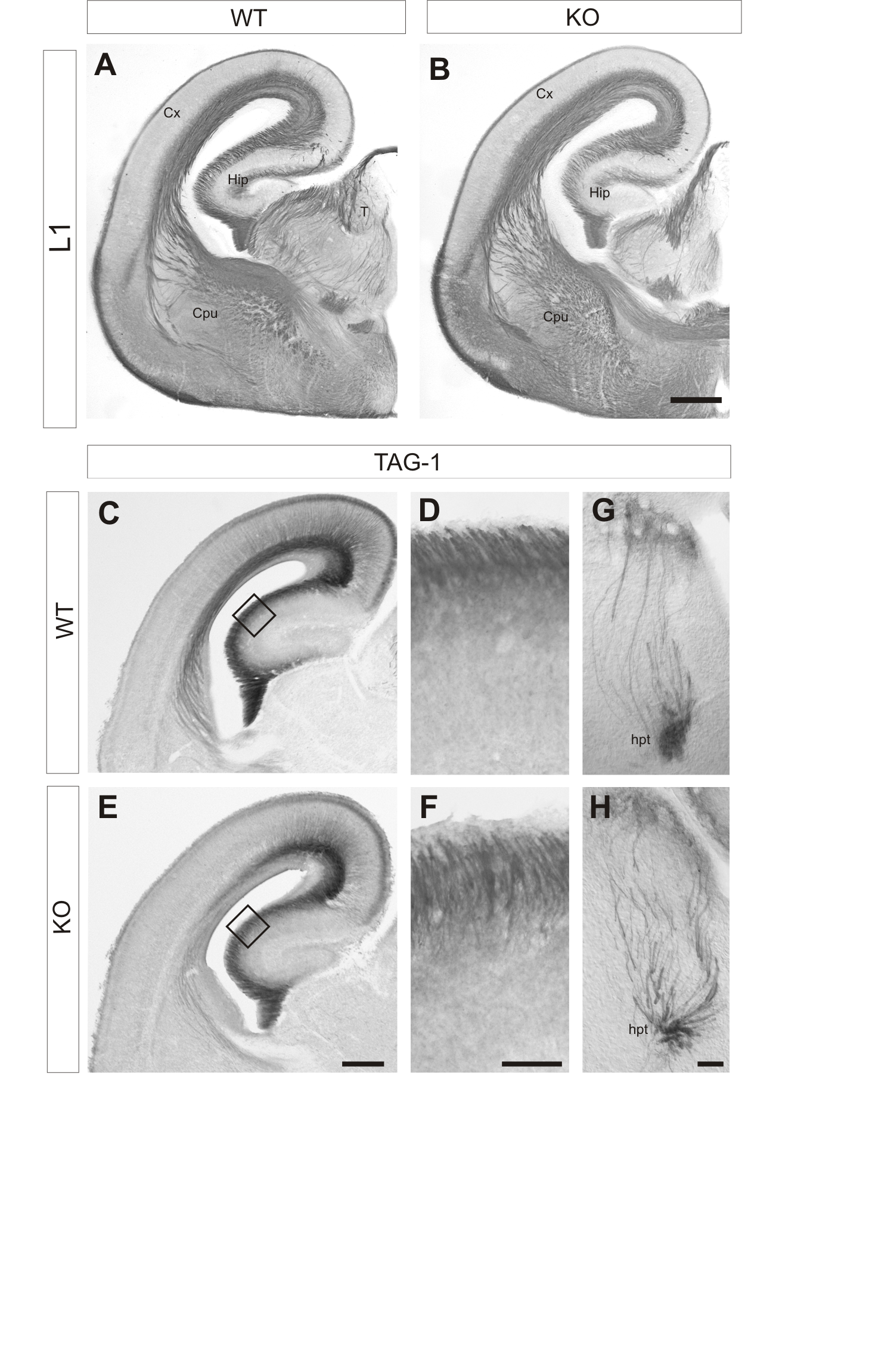

Supplement: Figure S3 — PC-deficient embryos display normal axonal trajectories but abnormal fasciculation. (A, B) Low-power views of L1-immunolabeled forebrain sections showing a normal distribution of fibers in podx(−/−) embryos, compared to wt brains at E18. (C–F), shape and size of TAG-1 immunoreactive axonal fascicles are altered in PC-deficient hippocampus. Note that in the podx(−/−) hippocampus (E, F) axonal bundles of the white matter were smaller, less compacted and occupied a wider zone in the adjacent stratum oriens, compared to wt littermates (C, D). (D, F) high magnification of C and E, respectively. (G, H) TAG1-immunoreacted sections showing increased defasciculation in the habenulo-peduncular tract of podx(−/−) embryos. Cx, cerebral cortex; CPu, caudate-putamen of the striatum; Hip, hippocampus; hpt, habenulo-peduncular tract; T, thalamus. Scale bars = 400 µm (A, B), 200 µm (C, E); 50 µm (D, F), 75 µm (G, H). (10.07 MB TIF) [file pone.0012003.s003.tif]

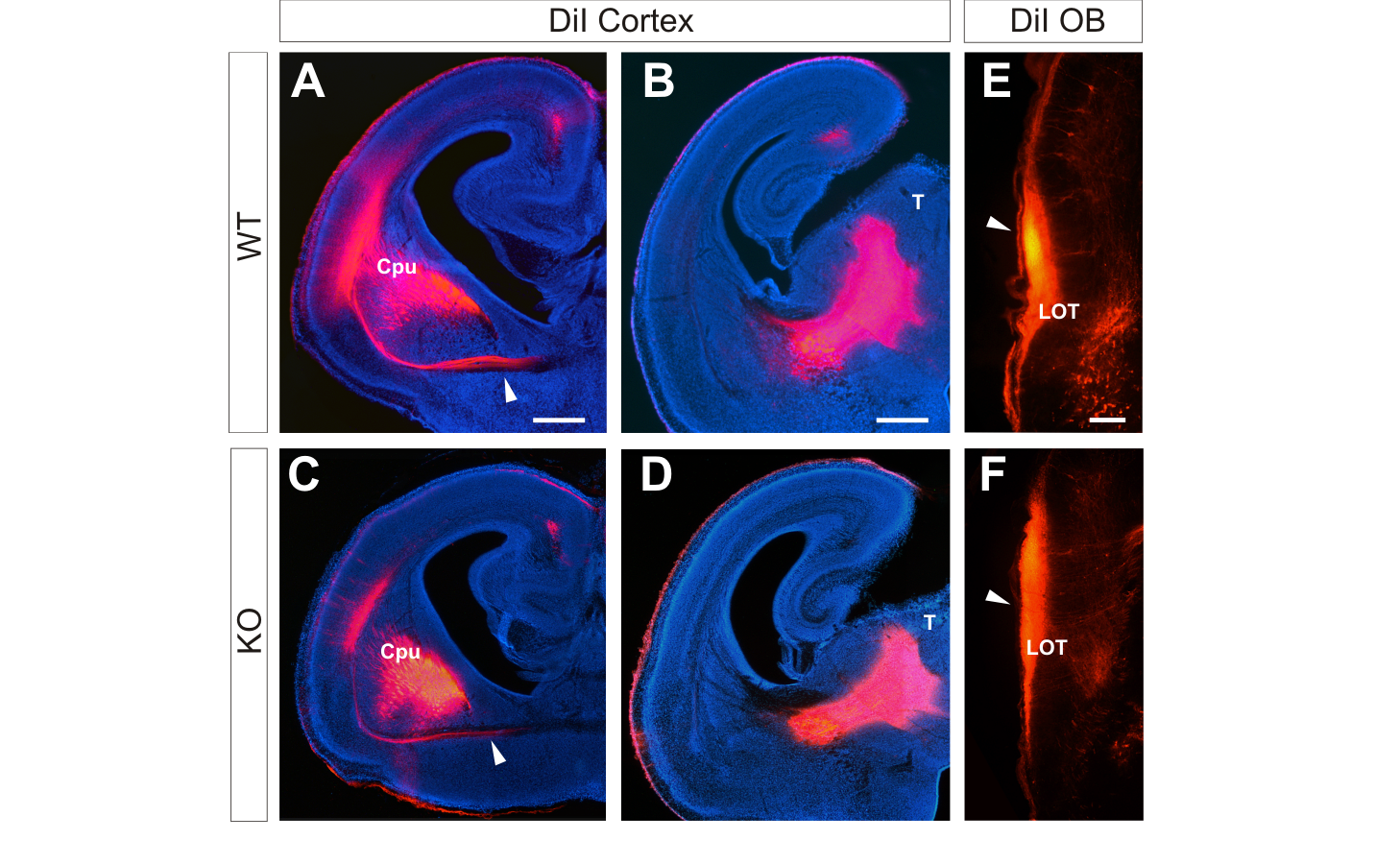

Supplement: Figure S4 — PC embryos display normal axonal projection patterns. Pattern of DiI labeling in wt (A,B,C) and podx(−/−) embryos (C,D,F) following DiI injections in the cerebral cortex (A,D) and olfactory bulb (E,F). After DiI injections in the cortex, robust corticothalamic projections invade the thalamus in wt (A,B) and podx(−/−) (D,E) embryos, traversing the striatum (Cpu). Arrowheads point to the anterior commissure. (C,F) Labeling of the Lateral Olfactory Tract after DiI injections in the Olfactory Bulb, suggesting normal formation of this tract in podx(−/−) embryos. All images correspond to coronal sections. Cpu, caudate-putamen; LOT, lateral olfactory tract; T, thalamus. Scale bars = 500 µm (A–D); 100 µm (E,F). (3.94 MB TIF) [file pone.0012003.s004.tif]
